# A renewal model for the emergence of anomalous solute crowding in liposomes

**DOI:** 10.1186/1752-0509-9-S3-S7

**Published:** 2015-06-01

**Authors:** Paolo Paradisi, Paolo Allegrini, Davide Chiarugi

**Affiliations:** 1 Istituto di Scienza e Tecnologie dell'Informazione "A. Faedo" (ISTI-CNR), Via Moruzzi 1, Pisa, Italy; 2 BCAM - Basque Center for Applied Mathematics, Alameda de Mazarredo 14, Bilbao, Basque Country, Spain; 3 Istituto di Fisiologia Clinica (CNR), Via Moruzzi 1, Pisa, Italy; 4 Department of Theory and Bio-Systems, Max Planck Institute of Colloids and Interfaces, Science Park Golm, Potsdam, Germany

**Keywords:** protein crowding, anomalous solute crowding, super-concentration, renewal processes

## Abstract

A fundamental evolutionary step in the onset of living cells is thought to be the spontaneous formation of lipid vesicles (liposomes) in the pre-biotic mixture. Even though it is well known that hydrophobic forces drive spontaneous liposome formation in aqueous solutions, how the components of the earliest biochemical pathways were trapped and concentrated in the forming vesicles is an issue that still needs to be clarified. In recent years, some authors carried out a set of experiments where a unexpectedly high amount of solutes were found in a small number of liposomes, spontaneously formed in aqueous solution. A great number of empty liposomes were found in the same experiments and the global observed behavior was that of a distribution of solute particles into liposomes in agreement with a inverse power-law function rather than with the expected Poisson distribution. The chemical and physical mechanisms leading to the observed "*anomalous solute crowding*" are still unclear, but the non-Poisson power-law behavior is associated with some cooperative behavior with strong non-linear interactions in the biochemical processes occurring in the solution. For tackling this issue we propose a model grounding on the Cox's theory of renewal point processes, which many authors consider to play a central role in the description of complex cooperative systems. Starting from two very basic hypotheses and the renewal assumption, we derive a model reproducing the behavior outlined above. In particular, we show that the assumption of a "*cooperative*" interaction between the solute molecules and the forming liposomes is sufficient for the emergence of the observed power-law behavior. Even though our approach does not provide experimental evidences of the chemical and physical bases of the solute crowding, it suggests promising directions for experimental research and it also provide a first theoretical prediction that could possibly be tested in future experimental investigations.

## Introduction

### Background and Motivations

In research concerning the origins of life, many efforts focus on understanding of the very first steps which led to the emergence of organic compounds and metabolic pathways in pre-biotic chemical solutions. A less investigated issue regards a crucial evolutionary stage, i.e., the spontaneous compartmentalization of both early biochemical reactions and related metabolites into cell-like structures. This is a crucial aspect, as high concentration levels of metabolites are needed to get a reasonable probability for the occurrence of biochemical reactions.

A widely accepted hypothesis assigns to semi-permeable lipid vesicles (liposomes) the role of hosting the very first metabolic pathways, thus acting as precursors of the living cells. A strong argument supporting this hypothesis comes from the well known behavior exhibited by (amphipathic) lipids when they are put in water solutions. In this setting, lipids aggregate to form liposomes, a spontaneous process driven by hydrophobic forces [1]. Even though the mechanism of vesicle formation is well assessed, a critical issue that needs to be clarified concerns the mechanisms by which components of metabolic pathways can reach concentration levels that can be much higher inside the vesicles than outside.

Simple liposomes cannot actively modify their content because they are not equipped with the structures (e.g. trans-membrane channels) needed for managing the exchange of solutes with the surrounding environment. Thus, the anomalous concentration levels, found in liposomes, of the components of the early metabolic pathways must have occurred as a spontaneous process, i.e. as the result of a chemical and physical self-organization. Actually, trans-membrane channels are also a manifestation of self-organization, but here we refer to a form of self-organization that is, in some sense, *simpler *or, in other words, more homogeneous in space and time, with respect to the complex structures emerging in the cell dynamics.

To address this issue, Luisi *et al*. [2-4] performed an elegant series of experiments consisting of the direct observation of the encapsulation of solutes inside lipid vesicles (liposomes) forming in an aqueous environment. These experiments produced surprising and intriguing outcomes regarding the spontaneous self-organization of vesicles containing an unexpectedly high amount of encapsulated molecules. In particular, the authors found that, when lipid surfaces close up in a solute-containing solution to form vesicles, the entrapment frequency does not follow the expected Poisson distribution, but tends to assume a power-law behavior, characterized by many empty vesicles (no or very few trapped molecules), and a long decreasing tail with extremely crowded vesicles. This effect has been denoted as "*superconcentration*" [2-4] and, in the following, will be referred to as "*anomalous solute crowding*".

In this paper we propose a modeling approach for gaining some insights into the physical mechanisms underlying these experimental results. In particular we show that describing the system in the framework of the *theory of renewal processes *[5], and applying two simple hypotheses, the experimental behavior observed by Luisi *et al*. [2-4] is straightforwardly reproduced.

In the next subsection we will provide a more detailed description of both the experimental setting and the "anomalous solute crowding" phenomenon reported in [2-4], underlining that the emergence of a power-law non-Poisson distribution involve a strong cooperative mechanism. We then proceed in the "*Methods*" section by reviewing the connection between renewal point processes and self-organized (cooperative) systems. In particular, the subsection "*Mathematical aspects of renewal theory*" is devoted to a brief sketch of the basic mathematical aspects of renewal point processes that will be used in the next sections. In the "*Results*" section we introduce our model and we discuss how the proposed model can help in shedding some light on the observations of Luisi *et al*. [2]. In the last section "*Conclusions*" we discuss the results, also suggesting new possible experimental validation of the model predictions.

### The experimental evidences

**Figure 1:**
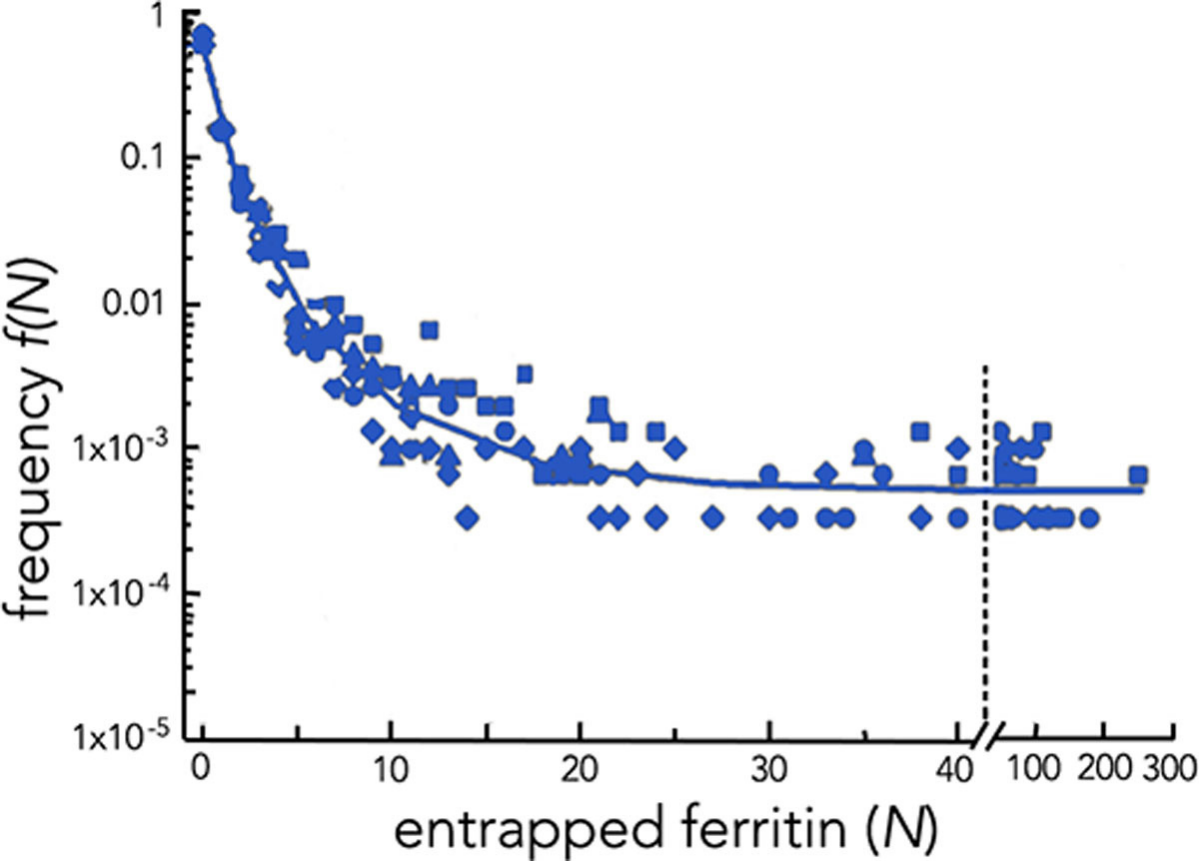
**Comparison between the expected Poisson distributions (open symbols) and the observed occupation frequency (closed symbols) of ferritin-containing liposomes plotted as theoretical or observed frequency versus the number of entrapped ferritin molecules per vesicle**. Concentration of ferritin solution: 4 (diamonds), 8 (circles), 16 (squares), 32 mm (triangles). Poisson curves refer to vesicles 100 nm in diameter. Data are plotted on a semilogarithmic graph. Note that Poisson distributions converge quickly to zero for high *N *. Redrawn from [2-4]

As sketched in the previous subsection, the experimental setting used by Luisi *et al*. [2] regards the formation of lipid vesicles in water solution containing a macromolecular solute. In particular, vesicles composed of 1-palmitoyl-2-oleoyl-snglycero-3-phosphati-dylcholine (POPC) or POPC/cholesterol were allowed to form in an aqueous solution containing soluble macromolecules such as ferritin. In this setting the liposomes, while forming, can trap the solute macromolecules. Once vesicles are formed, the encapsulated particles cannot flow out, due to the impermeability of the lipid membranes with respect to the water-soluble macromolecules. For the same reason, such macromolecules cannot cross liposome membranes from the outside. Thus, the amount of solutes present inside the vesicles after they have closed must necessarily have flown inside *before *the closure of the liposome membrane. Thus, the analysis of the liposome contents can be used to obtain realistic insights into the physics of solute encapsulation. In order to detect the molecular contents of each vesicle at the single molecule level, Luisi *et al*. used cryo-TEM, which is a very reliable method for imaging lipid vesicles. Cryo-TEM overcomes many limitations of the TEM instrumental technique, such as the presence of drying artifacts. Both ferritin molecules and ribosomes can easily be detected by cryo-TEM and counted individually and, thus, were used as solutes in the experiments. Unexpectedly, Luisi *et al*. noticed that lipid vesicles can spontaneously capture a very high number of macromolecular solutes, even when forming in diluted solutions. Thus, as a result of this unexpected behavior, a certain number of solute-rich vesicles, i.e., with anomalously high concentration levels of the solute, showed up in the chemical solution, and the overall distribution of solute particles trapped into liposomes was found to decay as an inverse power-law function (see Figure 1).

From a statistical point of view, the simplest assumption is that of a sequence of *independent *random trapping events of the solute molecules into the liposomes. Under this assumption, the entrapment of solute particles into liposomes of volume *V *(into a well stirred solution containing a solute *S *with concentration *C*_0_) can be considered equivalent to a series of independent random samplings of the *S *particles. It is then expected that, on average, each vesicle will contain *Nµ *= *NA V C*_0 _molecules, (with *NA *being the Avogadro number). Hence, under the assumption of independent trapping events and in absence of any other cooperative mechanism, the amount *N *of the solute *S *in the liposomes can be described by a Poisson distribution.

On the contrary, as shown in Figure 1, the direct observation of liposomes after solute entrapment revealed that the majority of vesicles (*>*80%) were empty, while about 0.1% *− *1% of vesicles contained a very high number of solutes (*N >*20, up to ca. 300), in clear contrast to the prediction of the Poisson law and in agreement with a power-law decay. This means that lipid vesicles can spontaneously capture a very high number of macromolecular solutes, thus producing an intra-vesicular concentration of particles that is significantly higher with respect to the surrounding aqueous solution. Noticeably, this "*anomalous solute crowding*" occurs also when lipid vesicles form in diluted solutions. This finding overcomes the problem arising in prebiotic chemistry of explaining how, despite the low environmental concentration of solutes, proto-cells might have enclosed in their aqueous core enough molecules for their metabolic needs.

The power-law non-Poisson profile of the solute distribution is a signature of cooperative dynamics. As the system is clearly characterized by critical closure events marking the final step of vesicle-forming cooperative dynamics, the use of stochastic point processes to describe this system seems to be a natural choice.

In the next section we discuss the renewal character of the point process describing the dynamics of ferritin trapping into forming lipid vesicles, trying to give a qualitative interpretation in terms of cooperation among different components of the aqueous solution: water molecules, lipids, macromolecules of solute (e.g., ferritin). We also sketch the general connection between complex cooperative systems and renewal events.

## Methods

### Renewal condition and cooperation

In the last few years renewal processes are becoming increasingly popular for describing important aspects of the behavior of complex systems [5-12]. These systems are characterized by the presence of cooperative or self-organized structures that emerge from the strong non-linearities of the underlying microscopic dynamics. Cooperative structures are coherent and meta-stable, i.e., they have relatively long lifetimes, during which the emerging structure maintains essentially unaltered some basic features that make the structure itself "*recognizable*", until a critical (shorttime) transition event occurs at some random time [13-15]. After that, a fast drop of the structure features (memory and spatial topology) occurs. Consequently, the renewal process can be identified as a birth-death process of cooperation, thus defining the sequence of transition events determining the decay of a coherent, long-lived structure and, possibly, the quasi-concurrent emergence of a new one.

A renewal process is rigorously defined as a sequence of events randomly occurring in time, without any dependence from the previous events or from external forces, and it is described as a sequence of event occurrence times {*t_n_*} (with *n ∈ N *^+^), enjoying the property that the Waiting Times (WTs) between two subsequent events *τ_n _*= *t_n _− t*_*n−*1 _are mutually statistically independent random variables.

In this work, the application and consequent interpretation of the renewal assumption is somewhat different with respect to the above described cases and closer to the definition of renewal process that can be found in the first pages of Cox's book [5]. Here the renewal process is again associated with a cooperative behavior, but the emergence of the self-organized structure is not associated with a transition event dynamically connecting one meta-stable structure to another. In the case under study, the cooperative interactions among lipids are given by the hydrophobic forces that give rise to a spheroidal self-organized structure, i.e., the *liposome*. The cooperative behavior extends also to the solute molecules. Each solute molecule interacts with the lipids during liposome formation and possibly with other solute molecules, in such a way that the number of solute molecules trapped in the liposomes are distributed according to an inverse power-law.

Notice that if there was no cooperation at all time and space scales, i.e., if there were no lipid-lipid interactions (liposome formation) neither ferritin-lipide and/or ferritin-ferritin interactions, totally independent events could be assumed. In this very simplified case, for any arbitrary chosen spatial volume, the statistical distribution of the number of both ferritin molecules and lipids in a given should be a Poisson one. As this is not the case, cooperation must emerge from some non-linear interactions among molecules.

Firstly, the "*total independence*" assumption is not compatible with liposome formation, which requires cooperation among lipid molecules, that is actually triggered by lipid-water interactions, i.e., by hydrophobic forces. Consequently, a first level of cooperation must be assumed and it is quite evident in the spontaneous emergence of structures such as liposomes.

However, the above observation is not sufficient to justify the anomalous solute crowding, which requires at least another cooperative force: a lipid-ferritin strong interaction or ferritin-ferritin (e.g., clustering) or both of them must be assumed.

Alternatively, the power-law distribution could be the consequence of an anomalous diffusion process of ferritin molecules inside water coupled with some clustering mechanism, which could depend only on ferritin itself or, on the contrary, on both ferritin and lipids. Note, however, that this hypothesis would again imply some kind of cooperation, but beetween ferritin and water, so that self-organization would yet emerge as a consequence of cooperative forces.

Here we limit to the first assumption, i.e., the lipid-ferritin interaction, and we show that this assumption is sufficient to derive the observed power-law decay in the overall distribution of trapped solute molecules.

It is important to underline that the model here proposed describes the average behavior of the system, without any explicit hypothesis on the chemical and physical microscopic mechanisms, but only resuming general principles on self-organized complex systems.

In the next subsection some mathematical definitions and results regarding renewal point processes are briefly reviewed. These results are exploited in the next section, where the proposed trapping model is introduced and discussed.

### Mathematical aspects of renewal theory

Here we briefly recall the basic mathematical concepts of renewal theory. The critical closing event separates the time axis into a pre-event time period with an evolving number of ferritin molecules *N *(*s*)*, s < τ *and a post-event period with a stationary, equilibrium, condition: *N *(*s*) = *N_∞_, s > τ *.

In renewal theory, the most important quantity is given by the statistical distribution of the survival (closing) times, i.e., the Survival Probability Function (SPF):

(1) $$\Psi \left(t\right)=Pr\left\{\tau >t\right\}.$$

and the associated Probability Density Function (PDF):

(2) $$\psi \left(t\right)dt=Pr\left\{t<\tau \leq t+dt\right\};\;\psi \left(t\right)=-\frac{d\Psi }{dt}\left(t\right)$$

In analogy with the Cox's failure rate, the rate of closing events is defined by the expected number of events per time unit in a neighborhood of the time *t *[5], also denoted as *local rate of event production r*(*t*). The time *t *is the time spent after an initial time *t*_0 _= 0 at which all the lipids simultaneously begin to cooperate to generate the different liposomes. In a more rigorous way, the rate *r*(*t*) is defined as the conditional probability density that a critical event occurs in an infinitesimal time interval [*t, t *+ *dt*], given that no events occurred in the time interval [0*, t*]:

(3) $$r\left(t\right)=\underset{dt\to 0}{\text{lim}}\frac{\text{1}}{dt}\text{Pr}\left\{t<\tau \leq t+dt|\tau >t\right\}.$$

It is important to note that *r*(*t*) is not a probability, neither a probability density. This is related to the definition involving the conditioning on the "survived" systems, *i.e*., not yet closed liposomes in our case. By using the properties of conditional probabilities: *P *(*A, B*) = *P *(*A|B*)*P *(*B*), being *A *= {*t < τ ≤ t *+ *dt*} and *B *= {*τ > t*}, we have:

$$\text{Pr}\left\{t<\tau \leq t+dt|\tau >t\right\}=\frac{Pr\left\{t<\tau \leq t+dt\right\}}{Pr\left\{\tau >t\right\}}=\frac{\psi \left(t\right)dt}{\Psi \left(t\right)},$$

where Eqs. (1) and (2) have been used. Substituting this expression into Eq. (3) it is easy to prove the following expression for the rate *r*(*t*) [5]:

(4) $$r\left(t\right)=\frac{\psi \left(t\right)}{\Psi \left(t\right)}=-\frac{\text{1}}{\Psi \left(t\right)}\frac{d\Psi }{dt}\left(t\right).$$

For a given rate *r*(*t*), this equation for Ψ is easily solved and it gives the following equivalent formulation:

(5) $$\Psi \left(t\right)=\text{exp}\left(-\int_{0}^{t}r\left(t^{\prime }\right)dt^{\prime }\right),$$

being Ψ(0) = *P r*{*τ >*0} = 1.

The SPF of a time-homogeneous Poisson process is an exponential function:

(6) $$\Psi \left(t\right)=\text{exp}\left(-r_{p}\;t\right),$$

so that, comparing with Eq. (5), it results that the event rate is constant in time: *r*(*t*) = *r_p _*[5]. This is in agreement with the well-known property that, in a Poisson process, the mean number of events in a given time interval [*t, t*+Δ*t*] is proportional to the length of the time interval itself: $\langle N_{p}\left[t,t+\Delta t\right]\rangle =r_{p}\Delta t.$

We note that a inverse power-law behavior can emerge only if the event rate is assumed to change in time and, in particular, if it has the following expression (see, e.g., [6]):

(7) $$r\left(t\right)=\frac{r_{0}}{\text{1}+\lambda t},$$

from which, using Eq. (5), it is easy to derive the following inverse power-law expression for the SPF:

(8) $$\Psi \left(t\right)={\left(\frac{\text{1}}{\text{1}+\lambda t}\right)}^{r_{0}/\lambda }$$

## Results

In this section we introduce the model assumptions and show in detail the development of the model and the derivation of the main result, given by the power-law distribution in the number of solute molecules trapped inside the liposomes.

### Model assumptions

The lipid-solute interaction, discussed in the previous Section, is encoded in the capacity of the liposome to trap solute molecules, i.e., in the flux rate of molecules entering into the forming liposome through its open borders. Given the reasonable assumption that the probability of trapping is proportional to the area of the open surface of the liposome (permeability), the flux rate of solute turns out to be proportional to the closing rate *r*(*τ *) of the liposome borders, being *τ *the time elapsed since the beginning of the liposome formation. The ferritin-lipid interaction hypothesis implies that the closing rate and, consequently, the ferritin flux rate, is affected by the passage of ferritin itself.

It is worth noting that the assumption of a ferritin-lipid interaction affecting the liposome closing rate is not incompatible with a possible anomalous diffusion of ferritin and/or lipid molecules inside water coupled with some clustering mechanism. However, this assumption needs a experimental validation and, consequently, the setup of new experimental work and will be the subject of future investigations.

We also note that, if the motion of solute particles in the aqueous solution was completely random, the net flux rate of solute on the liposome open borders would be zero. As a consequence, an asymmetric mechanism driving the flux rate must be assumed in order to get an average net flux of solute inside the liposome.

The asymmetric mechanism should involve a interaction ferritin-ferritin (clustering) and/or ferritin-lipid that changes depending on the position of the ferritin (inside or outside the forming vesicle). This asymmetry must determine a probability, for a ferritin molecule, of moving from the external environment into the vesicle greater than the opposite direction. Between these two hypotheses, the ferritin-lipid interaction might be more realistic. Indeed, soluble proteins such as ferritin do not clusterize spontaneously because of the composition of their surface which makes the interactions with water molecules thermodynamically more favourable than the protein-protein interaction.

In summary, our model is grounded on two hypotheses:

• The "*jamming hypothesis*" [2-4]: the interactions between the particles of solute and the incomplete vesicles interfere with (jam) the process of liposome formation; in particular the closing of liposomes is slowed.

• The "*semi-permeability*" hypothesis: the diffusion of solute particles from the solution towards the inside of the forming liposomes is faster than the diffusion in the opposite direction; in other words, there is a net flux of particles directed inside the forming vesicle.

These two hypotheses must be considered together with the renewal property, which is a reasonable assumption if each liposome does not interact with the other ones, that is:

(i) there are so many lipids that there's no competition in the formation of lipid vesicles or liposomes;

(ii) there are so many ferritin molecules and the liposomes are far enough from each other that there's no competition in the trapping of ferritin.

Then, the applicability of the renewal condition is exactly the same as in the Cox book [5] by substituting the *failure events *of some *electronic devices*, described there, with our *closing events *of some *liposomes*. In fact, under the assumptions (i) and (ii), the statistical ensemble of liposomes becomes a set of statistically independent realizations, so that both closing events and the associated closing times *τ *elapsed from the beginning of the experiment are mutually independent random variables and linear averaging can be applied to define mean quantities.

In the framework of renewal point processes, this model seems to be the minimal one explaining the emerging power-law behavior.

In fact, if we do not make this minimal assumption, the dynamics of lipids and ferritin would be independent and the flux of ferritin molecules across the liposome borders would be totally random, thus giving rise to a Poisson distribution of trapped ferritin molecules. Note that the renewal condition is associated with the emergence of an asymptotic self-organized structure (closed liposome + trapped ferritin molecules) following the occurrence of the closing event, i.e., the crucial event after which no flux of ferritin across the liposome surface is no longer possible. The renewal condition is then a natural hypothesis for the closing event, as the final state given by the asymptotic structure is well defined, but there's no way to know the exact time evolution that brought the system dynamics towards that particular asymptotic, stationary state.

In the next subsection we will show how, starting from these two very basic phenomenological hypotheses, it is possible to derive the observed power-law behavior.

### Derivation of the model

Given the assumptions and hypotheses stated above, we will deal with the problem of finding the form for the event rate *r*(*t*) which is consistent with the experimentally observed power-law behavior for the distribution of ferritin molecules trapped inside the liposomes.

The jamming hypothesis can be formalized as follows:

(9) $$r_{c}\left(t\right)=\frac{r_{0}}{\text{1}+N\left(t\right)},$$

being *N *(*t*) the number of ferritin molecules trapped inside the liposome at time *t*. This is almost a natural choice, as we require a rate function that must slow down when *N *(*t*) increases, while the unit in the denominator avoid singularities at *t *= 0. *r*_0 _is a dimensional constant representing the closing rate of the liposomes in absence of ferritin. *N *(*t*) is not a deterministic function, monotonically increasing with time, rather it is a fluctuating random variable with an average tendency to increase. In other words, the semi-permeability hypothesis imposes that the average number of ferritin molecules $\langle N\rangle \;$(*t*) is a increasing function of time *t*. The rate *r*_*c*_(*t*), given in Eq. (9), is also a random variable, as it depends on the random variable *N *(*t*). Essentially, *r*_*c*_(*t*) is the closing rate associated with the stochastic dynamics of a single liposome interacting with the surroundings solute molecules (ferritin and lipids), while, under the renewal assumption, the average rate $\langle r_{c}\rangle$(*t*) describes the average behavior of the total ensemble of independent systems. Each system is composed by a liposome approaching the closing event and by the surrounding ferritin molecules randomly passing through its surface.

In order to derive a formula for the average rate $\langle r_{c}\rangle$(*t*), we consider the following expression:

(10) $$N\left(t\right)=\langle N\rangle \left(t\right)+\Delta N\left(t\right),$$

where we introduced the zero-mean fluctuation Δ*N *(*t*). Let us assume, without any loss of generality, that *N *(*t*) is a Poisson counting process. Then, the following well known result applies:

(11) $$\langle {\left(\Delta N\right)}^{\text{2}}\rangle \left(t\right)=\langle N\rangle \left(t\right)\Rightarrow \Delta N\left(t\right)\sim \sqrt{\langle N\rangle \left(t\right)}.$$

Eq. (9) can be rewritten in the following way:

(12) $$r_{c}\left(t\right)=\frac{r_{0}}{\text{1}+\langle N\rangle \left(t\right)+\Delta N\left(t\right)}=\frac{r_{0}}{\text{1}+\langle N\rangle \left(t\right)}\frac{1}{\text{1}+\Delta N\left(t\right)/\langle N\rangle \left(t\right)},$$

For time *t *large enough, the ratio $\Delta N\left(t\right)/\langle N\rangle \left(t\right)$ becomes negligible due to Eq. (11), which involves $\Delta N\left(t\right)/\langle N\rangle \left(t\right)\sim \text{1}/\sqrt{\langle N\rangle \left(t\right)}.$ Then, we have:

(13) $$r_{c}\left(t\right)≃\frac{r_{0}}{\text{1}+\langle N\rangle \left(t\right)}\left(\text{1}-\frac{\Delta N\left(t\right)}{\langle N\rangle \left(t\right)}\right),$$

and, making the average of both sides, we finally get:

(14) $$\langle r_{c}\rangle \left(t\right)=\frac{r_{0}}{\text{1}+\langle N\rangle \left(t\right)},$$

with $\langle \Delta N\rangle \left(t\right)=0$ by definition. In summary, the average closing rate $\langle r_{c}\rangle$ of a given liposome depends on $\langle N\left(t\right)\rangle$, i.e., on the average number of solute particles present in the vesicle at time *t*. We note that, for the above derivation of Eq. (14) it is sufficient to assume a negligible fluctuation Δ*N *(*t*) for large times *t*, without resorting to the assumption of a Poisson process for *N *(*t*). On the contrary, the semi-permeability hypothesis is a fundamental one, as it allows to derive a simple linear expression for *N *(*t*). This hypothesis simply states that, while liposomes are forming, the flux of solutes per unit of time from the environment towards the inside of the vesicles (*λ_in_*) is greater than the flux in the opposite direction (*λ_out_*). It is reasonable to assume that the relative slowdown of the outward flux can be due to some interactions between the solute molecules and the lipids composing the inner face of the vesicle. Thus, there is a net flux of solutes per unit of time (*λ *= *λ_in _− λ_out_*) directed towards the inner part of the forming vesicle. As a consequence, making an additional, but reasonable, linear assumption, we get:

*< N *(*t*) *>*= *λt*, and Eq. (14) becomes:

(15) $$\langle r_{c}\rangle \left(t\right)=\frac{r_{0}}{\text{1}+\lambda t}.$$

This equation is formally identical to Eq. (7), so that the average Survival Probability Function of the liposome closing times is given by Eq. (8). The PDF describing the distribution of closure times is easily obtained by deriving Eq. (8):

(16) $$\psi \left(t\right)=-\frac{d\Psi }{dt}=\frac{r_{0}}{{\left(\text{1}+\lambda t\right)}^{\text{1}+r_{0}/\lambda }}$$

Note that both Eqs. (8) and (16) become a purely inverse power-law distributions in the limit of large times: *t >>*1*/λ *= *T *.

The distribution of ferritin molecules trapped inside liposomes: *P *(*N *), is computed by applying the relationship defining the probability distribution of the function of a random variable, which is a random variable itself. In this case, the random function is given by *N *= *N *(*τ *), being *τ *the closing times whose PDF is given by *ψ*(*t*). Then, approximating *N *with a continuous variable in the range of large *N*, we have:

(17) $$P\left(N\right)dN=\psi \left(t\right)dt,$$

and, substituting Eq. (16), we get the following expression:

(18) $$P\left(N\right)=\frac{\left(\mu -\text{1}\right)}{{\left(\text{1}+N\right)}^{\mu }},$$

being:

(19) $$\mu =\text{1}+\frac{r_{0}}{\lambda }.$$

**Figure 2:**
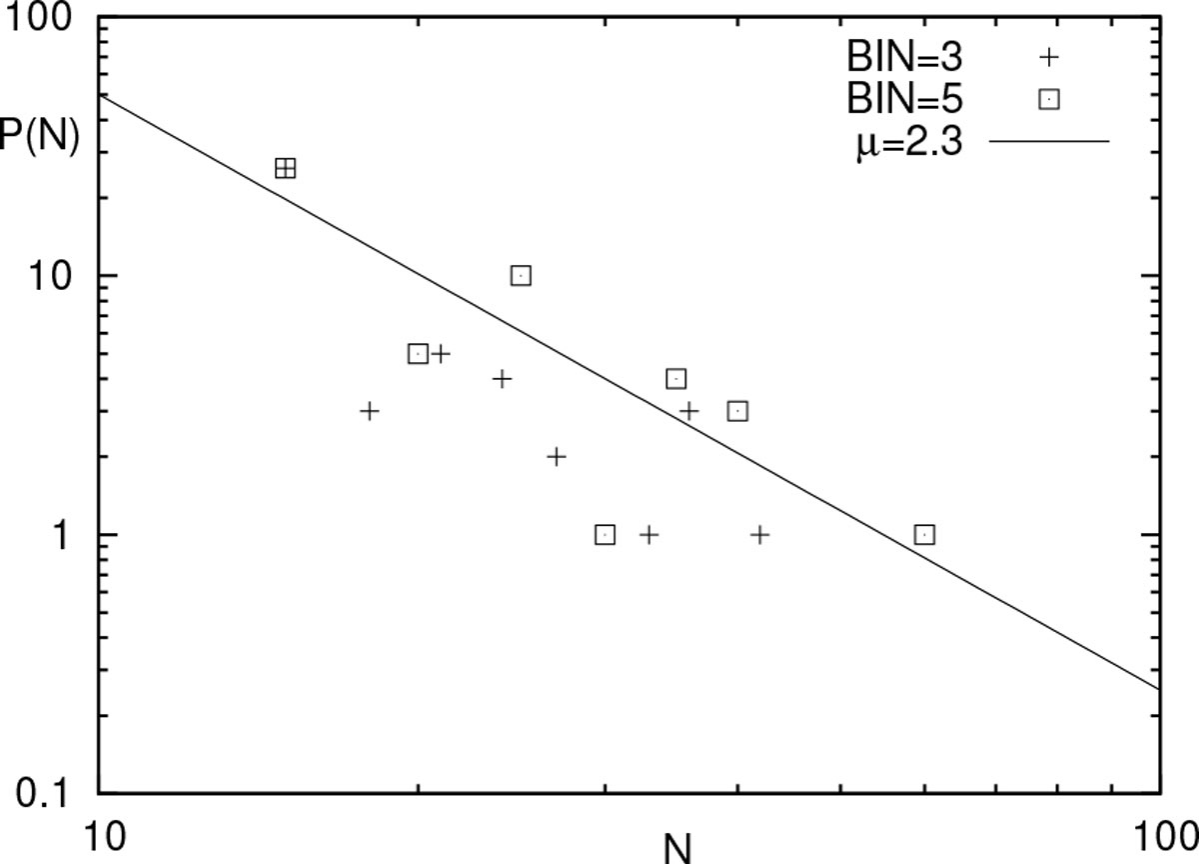
**Experimental data from Luisi *et al***. [2] are reported. Two different histograms are compared for different values of the binning (crosses: BIN = 3 and squares: BIN = 5). The continuous line is a guide-to-the-eye given as a pure power-law decay 1*/t^µ ^*with *µ *= 2.3 derived with a best-fit of Eq. 18 against experimental data.

The derivation of the expression for *P *(*N *) and the relationship given in Eq. (19), relating the exponent of *P *(*N *) with the parameters of the closing rate function *(r_c_)*(*t*) represent the main result of this work. As a preliminary validation, we made a fit with data taken from the paper of Luisi *et al*. [2]. Fitting Eq. (18) with the data, we obtain the best agreement for *µ *= 2.3, as shown in Figure 2.

## Conclusions

Starting from the experimental data reported in [2] we provided a model which reproduces the observed behavior, namely the so-called "super-concentration" effect, consisting in the anomalous crowding of solute inside a few lipid vesicles. The characterization of this phenomenon would be of paramount relevance for shedding some light on the very first steps which led to the formation of the earliest proto-cellular structures, which are supposed to be the ancestors of living cells. The physical bases underlying the spontaneous emergence of solute-rich lipid vesicles in aqueous solutions are still unexplained. Our findings suggest that the causes of the anomalous solute crowding could stem from the interactions between the lipids of the forming vesicles and the molecules of the solute. According to our model, these interactions are supposed to have two effects. On the one hand, they slow the closure of the liposomes (*jamming hypothesis*). On the other hand, the interaction between the solute and the inner surface of the liposome, slows the outflux of solute particles from the closing liposomes, thus making the inward flux greater than the flux outwards and giving rise to a net inward flux (*semipermeability hypothesis*). Recent experimental evidences [16] support the existence of spontaneous weak interactions between soluble proteins and lipid membranes that are supposed to arise from the fact that, in certain conditions, solute-lipids interactions can be thermodynamically more advantageous than the water-lipids interactions. Even if these findings are preliminary, they suggest some promising directions for experimental investigation. Furthermore in [17] it is shown that the interactions between proteins and lipid membranes have effects on the curvature of the bi-layers, thus suggesting a possible mechanism through which the jamming hypothesis may come into play.

We have also showed that, without the semipermeability hypothesis, the jamming hypothesis alone is not sufficient for giving rise to the observed steady-state powerlaw distribution of the solutes into liposomes. This finding is new in the literature regarding the super-concentration effect and may be a clue for experimental biologists aiming at better characterizing the nature of the lipid-solute interactions. Moreover, our model shows that the two hypotheses are sufficient for explaining the experimental observations. This implies that once the features and the dynamics of the lipid-solute interactions were be clarified, the observed anomalous crowding will automatically be explained. Clearly, it could be possible to make different assumptions and derive different models. As an example, it could be possible to give a different interpretation of the rate *r*(*t*), i.e., instead of a closing rate coming from a lipid-solute interaction a variable influx rate of solute molecules with constant closing rate. However, in this case, the renewal assumption, which is a natural choice in our modeling approach, cannot be applied. Then, a different relationship among the power index *µ *and the parameters of the rate *r*(*t*) would emerge, and also a different functional form of *P *(*N *) would be derived.

We believe that our approach, given the state of the art of the experimental knowledge, allows to explain the observed phenomenon with a minimal set of natural and plausible assumptions. These aspects deserve further investigations and, in particular, a close interaction with experimentalists in order to verify the theoretical prediction given by Eq. (19). A possible experimental verification could be obtained applying experimental techniques such as those proposed in [18], which allows us to measure the timing of the liposome closure process. In this setting, the jamming hypothesis could be straightforwardly verified comparing the closure rate of lipid vesicles with and without the presence of solute.

## Competing interests

The authors declare that they have no competing interests.

## Authors' contributions

This study was designed by all authors. All authors discussed the main ideas, the methods and the results. The paper was written by P.P. and D.C., with inputs from P.A. P.P. and D.C. analyzed the experimental data. P.P. and P.A. sketched the theoretical treatment. P.P. carried out most of the mathematical calculations.

## Declarations

P.P. acknowledges Collaborative European Project Grant N. 611516: "SEMEiotic Oriented Technology for Individual CardiOmetabolic risk self-assessmeNt and Self-monitoring (SEMEOTICONS)" and "CNR Short Term Mobility Program 2014" for financial support. D.C. acknowledges the FLAGSHIP Project "InterOmics" (PB.P05) that is funded and supported by the Italian Ministry of Education, University and Research, and by the National Research Council of Italy.

Publication costs for this article were funded by the above cited projects: European Project Grant N. 611516 (SEMEOTICONS) and FLAGSHIP Project "InterOmics" (PB.P05).

This article has been published as part of *BMC Systems Biology *Volume 9 Supplement 3, 2015: Proceedings of the Italian Society of Bioinformatics (BITS): Annual Meeting 2014: Systems Biology. The full contents of the supplement are available online at http://www.biomedcentral.com/bmcsystbiol/supplements/9/S3.
